# Hysterosalpingography using Magnetic Resonance Imaging for infertility patients

**DOI:** 10.5935/1518-0557.20210002

**Published:** 2021

**Authors:** Leandro Accardo de Mattos, Luísa Jacques Sauer, Roberto Blasbalg, Carlos Aberto Petta, Ricardo Mendes Pereira, Luiz Fernando Pina de Carvalho

**Affiliations:** 1 ALTA Excellency Diagnostic/DASA Department of Diagnostic Imaging/DASA, São Paulo Brazil; 2 Department of Diagnostic Imaging, Federal University of São Paulo, São Paulo, Brazil; 3 Fertilidade & Vida, Campinas Brazil; 4 Santa Joana Endometriosis Center, São Paulo Brazil; 5 Baby Center - Center for Reproductive Medicine, São Paulo, Brazil; 6 College - Institute of Clinical Research and Teaching Development, São Paulo, Brazil

**Keywords:** Hysterosalpingography, Magnetic Resonance Imaging, infertility, Image, Tubal factor Infertility

## Abstract

**Objective:**

Some studies have shown that it is possible to evaluate tubal permeability through MRI. Our aim is to perform a prospective study and to perform a comprehensive review in the literature regarding HSG-MRI.

**Methods:**

We carried out a PUBMED search using the following keywords: hysterosalpingogram, hysterosalpingography, magnetic resonance imaging and MRI. As inclusion criteria, we included only papers published in English, and exams ran on humans. We also conducted a prospective inclusion of patients who had visited a human reproduction clinic between May/2017 and April/2019 for laboratory image diagnoses using HSG-MRI.

**Results:**

Following the inclusion and exclusion criteria, we included seven original papers. Review papers and those written in a language other than English, were excluded. Between the period of May/2017 and April/2019, we selected ten patients for our study. The average exam duration was 30 minutes. Cervical catheterization was possible in all cases. There were no major complications. We highlight that in 8/9 of patients, we could directly visualize uterine tubes with contrast (excluding one patient with bilateral tubal ligation).

**Conclusions:**

Our initial experience with HSG-MRI shows promise. We demonstrated an optimized protocol for conducting an HSG-MRI (with excellent image quality). HSG-MRI had some advantages, such as not using ionized radiation, less pain and being able to analyze pelvic anatomy. Patients referred for a pelvic MRI as part of a more detailed investigation into infertility can also benefit from undergoing a simultaneous HSG using MRI.

## INTRODUCTION

The World Health Organization (WHO) estimates that 10 to 15% of women suffer from infertility and that, during diagnostic investigation, they undergo many laboratory-imaging exams (Carvalho *et al*., 2013). The main goals of these exams are to evaluate congenital genital tract anomalies, uterine, ovarian, and extrauterine disorders (such as endometriosis), as well as tubal diseases (Healy *et al*., 1994; Steinkeler *et al*., 2009).

In clinical practice, the principle modes of imaging to evaluate infertile women are hysterosalpingography using X-rays (HSG-XR), ultrasonography (US), hysterosonography, and pelvic magnetic resonance imaging (MRI) (Wall *et al*., 2015; Steinkeler *et al*., 2009). HSG-XR is the most used technique, and it is the gold standard for evaluating tubal permeability (Krysiewicz, 1992). However, this exam offers a limited evaluation of other uterine and extrauterine disorders that may be associated with infertility.

MRI on the other hand, with its high spatial resolution, contrast and capacity for multiplane reconstruction, enables an excellent characterization of a large variety of pelvic diseases, including those related with infertility, such as Mullerian anomalies (Behr *et al*. 2012; Marcal *et al*., 2011; Mueller *et al*., 2007; Pellerito *et al*., 1992; Scarsbrook & Moore, 2003; Troiano & McCarthy, 2004), adenomyosis (Tamai *et al*., 2005), leiomyomas (Deshmukh *et al*., 2012; Steinkeler *et al*., 2009; Woodward *et al*., 1993), inflammatory pelvic disease (Tukeva *et al*., 1999), and endometriosis (Imaoka *et al*., 2003; Novellas *et al*., 2010; Siegelman & Oliver, 2012). However, MRI is not able to evaluate whether or not the uterine tubes are obstructed, nor to provide detail regarding their aspect. We are only able to determine if there is tubal dilatation.

Some studies have shown that it is possible to evaluate tubal permeability through MRI utilizing the same technique as HSG-XR, using a saline solution with gadolinium instead of iodine contrast (Volondat *et al*., 2019; Sadowski *et al*., 2008; Unterweger *et al*., 2002; Wiesner *et al*., 2001; Winter *et al*., 2010). For those patients who had been recommended for a pelvic MRI for a more detailed infertility study, a simultaneous hysterosalpingography using the same method (HSG-MRI) enabled a single and complete exam to obtain all of the information associated with separate HSG-XR and pelvic MRI exams.

The main goal of our study was to review the literature regarding HSG-MRI, and to show our initial experience with ten patients submitted to this exam at our clinic, and to co-relate these results with those from the HSG-XR.

### Literature Review (Table 1)

**Table 1:** Summary of literature review data.

Author	Year	Patient sample	Equipment field	Contrast media	Contrast volume	Exam duration	Pain assessment	Analyzed variables (tubes)	Direct tubal visualization	Complications	MR HSG vs xR SG
Wiesner *et at*.	2001	5	1.5 T	Magnevist (gadopentetic ac. I Gd-OTPA)	15ml	55-70min	Yes	Patency	No	No report	MR Superior
Unterweger *et at*.	2002	10	1.5 T	Ootarem + Polyvidona	20ml	No report	Yes	Patency	Yes	No report	MR Superior
Sadowski *et at*.	2008	17	1.5 T	gadodiamide (omniscan) + saline (1:100)	2040ml	60 min	No	Patency	No	No report	NON
Winter *et at*.	2010	37	1.5 T	Ootarem (gadotericac)+ polyvidone	20ml	aprox 45 min	Yes	Patency	Yes	No report	MR Superior
Cipolla *et at*.	2015	116	3T	Gadopentetate dimeglumine (multihance) +saline (1:20)	4-5ml	aprox 18 min	Yes	Patency	Yes	No report	MR Superior
Kohan *et at*.	2017	22	1.5 T	Gd + saline (1:100)	average 26ml	average 49min	Yes	Patency	Yes	2 bleedings	MR Superior
Volondat *et at*.	2018	40	1.5 T	Ootarem diluted in SF 1:14	up to 8 ml	No report	Yes	Patency	No	4 pain cases	MR Superior

To find the papers included in this study, we carried out a PUBMED search using the following keywords: *hysterosalpingogram, hysterosalpingography, magnetic resonance imaging and MRI* (search index attached). As criteria, we included only papers published in English and exams that were carried out on humans. We evaluated each study, when pertinent, by: main goal, patient sample, study design, technical aspects related to HSG-MRI image acquisition (equipment field, cervical catheterization, form of contrast injection, dynamic sequence, and contrast used during the exam), tolerability and pain during the exam, analysis of image variables in the tubal evaluation to verify comparison with the gold standard method and result accuracy, and aggregated value from the MRI in characterizing extra tubal alterations, among others.

Following the inclusion and exclusion criteria of the 13 thousand papers found, we looked at seven original papers. Review articles and those written in a language other than English, were excluded. All articles sought to evaluate the viability of an HSG-MRI in determining tubal patency (Wiesner *et al*., 2001; Sadowski *et al*., 2008; Unterweger *et al*., 2002; Winter *et al*., 2010; Cipolla *et al*., 2016; Pelegrí Martínez *et al*., 2017; Volondat *et al*., 2019).

Five of the seven studies used the gold standard for comparison: four used an HSG-XR (Wiesner *et al*., 2001; Unterweger *et al*., 2002; Sadowski *et al*., 2008; Volondat *et al*., 2019) and one used chromopertubation (Pelegrí Martínez *et al*., 2017). There were similar findings between the methods in 73% to 100% of the cases in these studies. It is worth noting that Sadowski *et al*. (2008) showed a higher number of pervious tubes using HSG-MRI than using HSG-XR. In addition to a comparison between the methods, Volondat *et al*. (2019) also evaluated the inter- and intra-observer agreement in the analysis of HSG-MRI images with a kappa of 0.76 (substantial agreement) and 0.92 (almost perfect agreement), respectively.

Looking at the other two studies that did not use the gold standard, one analyzed HSG-MRI images in agreement by two observers (Cipolla *et al*., 2016) and the other evaluated inter-observer agreement (Winter *et al*., 2010), which was 100%. Six of the studies were conducted using a 1.5 Tesla equipment (Wiesner *et al*., 2001; Unterweger *et al*., 2002; Sadowski *et al*. (2008); Winter *et al*., 2010; Pelegrí Martínez *et al*., 2017; Volondat *et al*., 2019) and only one using a 3 Tesla machine (Cipolla *et al*., 2016).

The types of contrast used (all gadolinium based) varied among the studies and included: gadopentetic acid (Magnevist), gadoteric acid (Dotarem), gadodiamide (Ominscan), gadopentetate dimeglumine (Multihance). These contrast mediums were diluted in various proportions in saline solution. Winter *et al*. (2010) and Unterweger *et al*. (2002) opted to use a combination of the contrast medium with povidone in order to increase the solution’s viscosity.

In four of the studies, the contrast was injected using a pump (Wiesner *et al*., 2001; Winter *et al*., 2010; Pelegrí Martínez *et al*., 2017; Volondat *et al*., 2019), in two, the injection was manual (Cipolla *et al*., 2016; Sadowski *et al*., 2008), and one study did not specify it (Unterweger *et al*., 2002). The average duration of the exam varied from 18 to 70 minutes. Cipolla *et al*. (2016) used a 3 Tesla machine, and reported the shortest exam time with an average duration of 18 minutes. Two studies did not report exam durations (Unterweger *et al*., 2002; Volondat *et al*., 2019). Only Unterweger *et al*. (2002), Winter *et al*. (2010), and Cipolla *et al*. (2016) reported considerable rates of direct visualization of the tubes, specifically in: 5/7 (71%), 27/37 (73%) and 76/112 (68%) of the patients, respectively. The studies published by Volondat *et al*. (2019), Pelegrí Martínez *et al*. (2017), Sadowski *et al*., (2008), and Wiesner *et al*. (2001) did not document direct visualization of the tubes in the majority of the cases, which limited tubal evaluation to an investigation of patency inferred from extravasation into the peritoneal cavity, and provided no more anatomical detail.

In addition to studying tubal perviousness, Cipolla *et al*. (2016) analyzed the diameter of the tubes and contrast dispersion in the peritoneal cavity. Volondat *et al*. (2019) created a classification that considered perviousness, velocity, and symmetry of the contrast extravasation; and they evaluated the relationship between the tubes and the ovaries. The other studies primarily reported tubal perviousness.

The exam was successful in the majority of the cases, in all of the studies. Unsuccessful cases occurred due to catheterization failure, catheter dislocation, motion artifacts, and interruption of the exam due to pain and claustrophobia, and genital tract malformations. In all studies, catheterization was conducted in a room different from the MRI, with the patient transported to the exam table afterwards. The patients were cannulated prior to the beginning of the exam in all cases, with the exception of Sadowski *et al*. (2008), who initiated the exam without the catheter and inserted it only for readings in the dynamic phase. With the exception of Sadowski *et al*. (2008), who did not evaluate pain and/or tolerability, the other studies highlighted good acceptance of the exam and in general, less discomfort in relation to the HSG-XR.

The adverse incidents related to the procedure were two small bleedings reported by Pelegrí Martínez *et al*. (2017); two cases of intense pain that evolved with vasovagal symptoms, and one of salpingitis, reported by Volondat *et al*. (2019). There were no reports of serious complications. Five of the seven studies (Sadowski *et al*., 2008; Winter *et al*. (2010); Cipolla *et al*., 2016; Pelegrí Martínez *et al*., 2017; Volondat *et al*., 2019) highlighted the additional value of an MRI, not only in the characterization of already known gynecological variations, but also in identifying new findings such as: leiomyomas, Mullerian malformations, endometriosis, micro-polycystic or atrophied ovaries, and pelvic adherences. Pelegrí Martínez *et al*. (2017), who used video laparoscopy with chromopertubation as the gold standard, also observed a good correlation between MRI and intra-operatory findings with the exception of pelvic adherences, which were underestimated in image evaluation.

## MATERIALS AND METHODS

### Case cohort

We conducted a prospective inclusion of patients who visited a human reproduction clinic between May/2017 and April/2019 for laboratory image diagnoses through HSG-MRI exams. The patients included were those who had a clinical infertility diagnosis (primary or secondary), had been recommended for an MRI due to infertility, and who had had an HSG-XR in the last six months. For inclusion in the study, the subject had to have both exams. The exams were carried out using a 3 Tesla machine (*MAGNETOM Skyra, Siemens Medical Solutions*^®^) with a surface coil.

**Figure 1:**
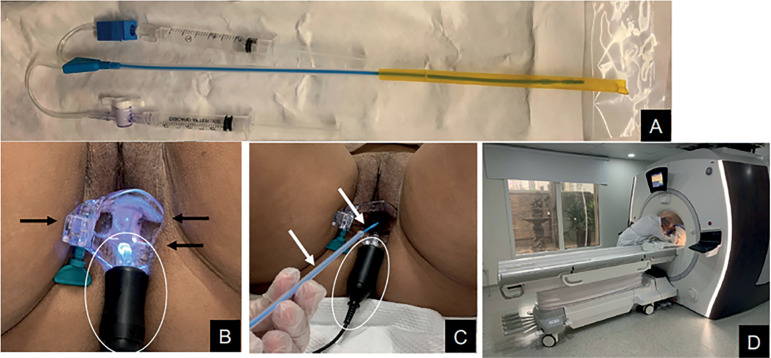
MR-HSG equipment **A:** MR-HSG equipment. Black circle: end of the catheter with the balloon that will be placed in the uterine cervix. Blue circle: syringe used to inflate the catheter. Yellow: syringe used to inject the contrast medium (gadolinium + saline). **B** and **C** illustrate items used: black arrows indicate the plastic speculum (MR compatible). The white circle shows the light connector for better view of the external orifice of the uterine cervix. White arrows show the HSG equipment as it is introduced in the vagina. **D:** patient positioned at the MR table as the radiologist administers the contrast medium.

We can divide our protocol into three distinct steps with the MR-HSG equipment, as per shown in Figure 1.

Step 1: Initially, we ran T1 and T2 weighted sequences for a routine evaluation of the pelvic structures, without venous administration of paramagnetic contrast - duration of approximately 15 minutes. 

Step 2: Subsequently, on the same examining table, cervical cannulation was performed using a 5F balloon catheter connected to specific HSG equipment. Between 15 and 20 milliliters of a solution composed of gadolinium (Dotarem^®^), diluted in 0.9% saline solution (1:100), administered manually during multiphase readings of T1 weighted dynamic sequences with fat suppression (with high temporal resolution), on the axial plane. We obtained 20 readings, with the objective of making a precise evaluation of the morphology and flow dynamics of the contrast through the uterine cavity, the Fallopian tubes, and its peritoneal dispersion. Immediately afterwards, we conducted a VIBE sequence with high spatial resolution - duration of approximately three minutes. 

Step 3: The patient was removed from the exam table, allowed to walk freely, and after a period that averaged 15 minutes (corresponding to the Cotté test of a conventional HSG-XR), the subject returned to the exam table to obtain the final weighted T1 sequence with fat suppression (high resolution spatial VIBE with a duration of approximately 30 seconds), in order to check the contrast dispersion into the peritoneal cavity and the retention of residual contrast in the tubes. When there was still contrast in the uterine cavity, we repeated this step after another walk by the patient. 

We adjusted the exam protocol during the study, especially with respect to the dynamic sequence in order to obtain better spatial resolution. Table 2 depicts the protocol for the optimized MRI after all adjustments.

**Table 2:** Optimized MR-HSG protocol. (FOV and MATRIX numbers it the table represent average values; some adjustments were made according to each patient's biotype).

Study phase	Sequence	Plane	RT(ms)	ET (ms)	FOV (cm)	Thickness (mm)	Matrix
1.Unhanced	3D-SPACE T2	Coronal	1200	112	36.0 x 57.7	1	384 x 384
	T2	Axial	8520	135	24.0 x 38.5	4	640 x 640
	T1 fat saturated (VIBE)	Axial	4.57	1.46	33.3 x 52.9	2	260 x 320
	T1 fat saturated (VIBE)	Sagittal	3.06	1.23	26.0 x 41.7	3	320 x 260
2. Dynamic with intrauterine contrast injection	VIBE (perfusion)	Axial	4.08	1.23	26.0 x 41.7	2	384 x 384
	Early post contrast	Axial	3.71	1.69	33.0 x 52.9	1.3	256 x 256
3.Cottê (late phase)	T1 GRE fat saturated (VIBE)	Axial	4.57	1.46	32.0 X 51.3	2	260 x 320

In order to reduce the effects resulting from intestinal peristalsis, an intravenous anti-spasm drug (1 ml of Buscopan^®^ 20 mg/ml) was administered in two doses; immediately prior to the beginning of the exam and again before the endocervical injection of the liquid. 

We analyzed the images at a workstation with a communication and image filing system (*Carestream PACS*) by a radiologist (LAM) with more than ten years of gynecological image experience. We searched for uterine and extrauterine alterations related to infertility (such as micro-polycystic ovaries, Mullerian malformations, endometriosis, adenomyosis, leiomyomas, polyps, pelvic adherences, etc.). With respect to the tubes, there were the following characteristics: dilation, perviousness, adherence, symmetric or hindered emptying of the contrast, retention of the contrast later in the sequence, and contrast dispersion in the peritoneal cavity. 

The same radiologist analyzed the HSG-MRI and HSG-XR exams, in a non-blinding way. 

## RESULTS

**Figure 2:**
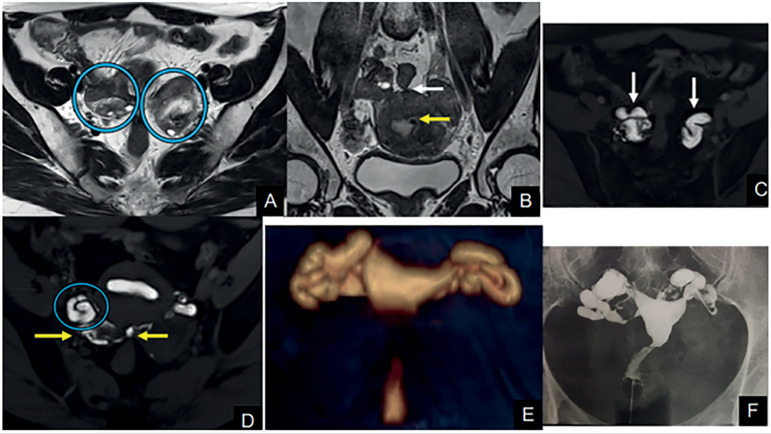
Exam example **A:** MR T2WI in the axial plane. The circles show ovaries close to the midline in the posterior pelvic pouch and adhered to the retrouterine endometriotic tissue. **B:** T2WI in the coronal plane. The yellow arrow shows a small submucosal fibroid measuring 3 mm; the white arrow indicates retrouterine endometriosis with adhesions involving both the ovaries and the rectum. **C:** Dynamic MR-HSG sequence in the axial plane. White arrows show bilateral hydrosalpinx with mucosal thickening. **D:** The circle shows right tube accumulated in the posterior pelvic pouch. **E:** MR-HSG volume rendering showing bilateral hydrosalpinx. **F:** Conventional HSG with bilateral hydrosalpinx.

**Figure 3:**
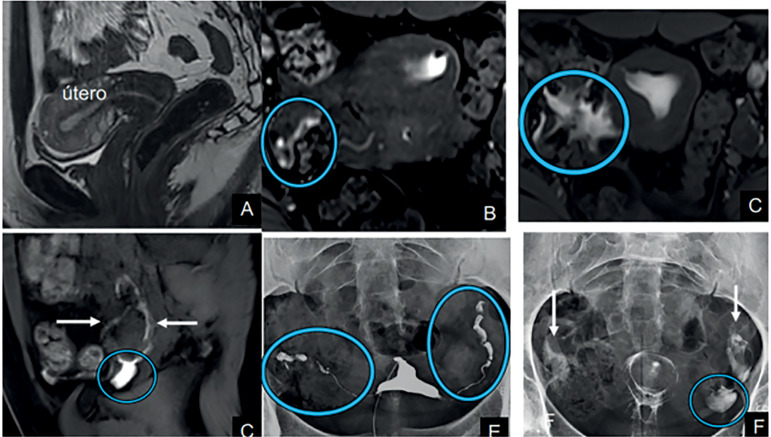
Exam example **A:** T2WI in the axial plane showing no abnormalities. **B:** Dynamic MR-HSG in axial plane. The circle shows part of the right tube, normal appearing. **C:** Dynamic MR-HSG axial plane. The arrow shows the uterine cavity filled with contrast and the circle indicates free tubal spillage to the peritoneal cavity. **D:** Dynamic MR-HSG in the sagittal plane. The arrow demonstrates the left ovary with a small amount of adjacent fluid. The circle shows a small amount of fluid in the left ovarian fossa. **E:** Conventional HSG showing normal tubes (circles). **F:** Conventional HSG, late phase (Cotté). The arrows demonstrate normal contrast dispersion in the peritoneal cavity. The circle shows periovarian fluid.

Between the period of May/2017 and April/2019, ten patients were selected for our study. Their demographic characteristics and HSG-MRI and HSG-XR image findings are available in Table 3. Figures 2 and 3 give examples of the exams.

**Table 3:** Demographic characteristics and imaging findings of our case cohort.

Patients	Age (years)	Exam indication	Infertility time (months)	Infertility type	Extratubal abnormalities - MR	Extratubal abnormalities - xR	abnormalities on xR-HSG	abnormalities on MR-HSG
1	32	Infertility check up	14	Primary	Polycystic ovaries	No	Small bilateral contrast retention	No
2	35	Pre Fertilization	36	Primary	No	No	No	No
3	34	Infertility	18	Secondary	No	No	Bilateral dilatation and obstruction with late contrast retention	Bilateral dilatation and obstruction
4	35	Infertility	24	Primary	No	No	Small contrast retention and pelvic adhesions on the left	Small contrast retention and pelvic adhesions on the left
5	35	Recurrent abortion		Primary	Small submucosal fibroids	Small submucosal fibroids	No	No
6	34	Treatment failure	24	Primary	Low follicle reserve (only 2 antral follicles altogether)	No	Small bilateral dilatation with late contrast retention	Small bilateral dilatation with late contrast retention
7	39	Infertility check up		Primary	Small submucosal fibroid, endometriosis, pelvic adhesions with posterior pelvic pouch blockage	No	Bilateral dilation, with patent tubes and diffuse adhesions	Bilateral dilation, with patent tubes and diffuse adhesions
8	33	Post tubal ligation follow up	120	Secondary	No	No	Bilateral tubal ligation, with obstructed tubes; no dilation	Bilateral tubal ligation, with obstructed tubes; no dilation
9	29	Infertility	12	Primary	No	No	Left periadnexal adhesions	Left periadnexal adhesions
10	42	Infertility check up	24	Primary	Adenomyosis, endometriosis, pelvic adhesions with posterior pelvic pouch blockage	No	Adhesions	Mild dilatation with late contrast retention and pelvic adhesions

The average exam length was 30 minutes (from the cervix catheterization onset until the end of the dynamic phases). In all cases, cervical catheterization was possible. We had one case of intense pain immediately after exam termination (probably due to chemical peritonitis), which was treated conservatively. There were no other complications. We highlight that in 8/9 patients, we could directly visualize uterine tubes with contrast (excluding one patient who had undergone bilateral tubal ligation).

In our study, the MRI showed extra-tubal pelvic alterations in five of the ten patients (50%). In only one patient, we found a submucous myoma by both methods. Another four (40%) showed extra-tubal alterations that were identified only by MRI; one with a small submucous myoma and endometriosis, one with adenomyosis and endometriosis, one with micro-polycystic ovaries, and another with signs of low follicular reserve (only one antral follicle in each ovary). In the patients with endometriosis, the pelvic adherence process was also better characterized by the MRI, with a more precise identification of vaginal sac blockage and adhered structures.

When we analyzed only the tubes and the contrast dispersion in the peritoneal cavity during the HSG-MRI phases and compared this to the HSG-XR, we noticed the aspects of the image were very similar in both methods. Of the ten patients, two (20%) had exam results that were considered normal by the HSG-MRI and the HSG-XR. The other eight patients (80%) showed some alteration that was found during this stage. One showed a ligation, with both methods showing the tubes to be completely occluded and with no other associated tubal alteration. In three patients, the HSG-MRI and the HSG-XR showed dilated tubes, with only one patient having a complete bilateral obstruction, and no leakage into the cavity (both methods demonstrated this finding). In the other two, both methods showed that the tubes were pervious, with adherences, and one of the patients showed liquid retention in the tubes during the last phase.

Two patients did not show tubal dilation; both the HSG-MRI and the HSG-XR showed exactly the same findings: one with liquid retention in the left tube, associated with an adherence process on this side and the other, only an adherence process located in the region of the left adnexal region, but with no tubal alterations.

In just two patients (20%), the HSG-MRI and the HSG-XR did not precisely show alterations. In one, the HSG-XR showed a small amount of liquid retention in the tubes during the late phase (not identified in the HSG-MRI). In the other patient, there were only adherences with no tubal alterations in the HSG-XR, while in the HSG-MRI; we identified a small bilateral tubal dilation associated with liquid retention in the late phase, as well as adherences.

## DISCUSSION

Tubal alterations can be identified in 20 to 36% of women investigated for infertility (WHO 1992; Healy *et al*., 1994), which has implications for clinical management (NICE Guidelines, 2013) and therefore, evaluation of the uterine tubes is indispensable. Traditionally, the principle imaging exam for analyzing tubal permeability in infertile women has been the HSG-XR, in spite of this method bearing some disadvantages such as radiation exposure, use of iodine contrast, low contrast resolution, and limited evaluation of other pelvic structures (such as the ovaries), as well as alterations located beyond the tubal and uterine lumen which could be associated with infertility.

MRI, in addition to not using ionizing radiation (therefore being innocuous to the female genital tract), is one of the most accurate methods for evaluating the pelvis, including in the context of infertility (Behr *et al*. 2012; Marcal *et al*., 2011; Mueller *et al*., 2007; Pellerito *et al*., 1992; Scarsbrook & Moore, 2003; Troiano & McCarthy, 2004; Tamai *et al*., 2005; Deshmukh *et al*., 2012; Steinkeler *et al*., 2009; Woodward *et al*., 1993; Tukeva *et al*., 1999; Woodward *et al*., 2001; Imaoka *et al*., 2003; Novellas *et al*. 2010; Siegelman & Oliver, 2012).

Over the last decades, studies have pointed to the viability of investigating tubular patency using MRI and all the additional diagnostics that MRI can provide in the evaluation of the uterine cavity and tubes (Volondat *et al*., 2019; Sadowski *et al*., 2008; Unterweger *et al*., 2002; Wiesner *et al*., 2001; Winter *et al*., 2010).Despite this, we have not seen a consistent use of this hybrid method in clinical practice.

Recognizing the promising role that the HSG-MRI can bring to the evaluation of infertile women, we ran a literature review of what has already been published about the method - which we believe to be the first in the literature dedicated exclusively to this topic. We also used our own experience with ten patients submitted to an HSG-XR and who underwent an HSG-MRI in our clinic, which enabled us to make a correlation between the two exams. We had the specific goal of optimizing technical parameters with the intent to obtain a better relationship between spatial and temporal resolution, and potentially achieving greater diagnostic performance.

We ran all our exams using a 3.0 Tesla machine, which we believe contributed to the direct visualization of the tubes with greater definition in almost all the cases. (8 of 9 cases). To our knowledge, there was only one previous test (Cipolla *et al*., 2016) using the 3.0 Tesla machine; the others were carried out using a 1.5 Tesla equipment. Carrascosa *et al*. (2016) in their review article, recommended the 3.0 Tesla field for the morphological evaluation of the tubes, but did not present a case analysis comparing the different magnetic fields.

The studies published by Volondat *et al*. (2019), Pelegrí Martínez *et al*. (2017), Sadowski *et al*. (2008), and Wiesner *et al*. (2001) evaluated the viability of the HSG-MRI using the 1.5 Tesla equipment. In these studies, it was not possible to see the contrast inside the tubes in most of the cases, which limited the evaluation to only an investigation of patency (inferred by the leakage of contrast to the peritoneal cavity) but without greater morphologic characterization of the tubes (Volondat *et al*., 2019; Wiesner *et al*., 2001; Pelegrí Martínez *et al*., 2017; Sadowski *et al*., 2008). Unterweger *et al*. (2002) and Winter *et al*. (2010) also ran their studies using a 1.5 Tesla machine; however, they were able to visualize the tubes in only 71% to 73% of their respective patients using a contrast medium with greater viscosity. Cipolla *et al*. (2016) used the 3.0 Tesla field and achieved direct tubal view in 67.8% of their patients. In their review, Carrascosa *et al*. (2016) suggested a preference for exams using machines with a 3.0 Tesla field; however, they did not present data comparing results between the 1.5 and 3.0 Tesla machines.

Volondat *et al*. (2019) emphasized the need for improving techniques that would provide greater anatomic detail of the tubes. We believe that we have achieved this objective with the technique that we used in our initial experiment. In eight of the nine cases, we visualized the tubes and described their morphology.

In addition to tubal permeability, we also evaluated the presence of dilation, tubal retention of the contrast in the late sequence, and its dispersion in the peritoneal cavity. In the literature review that we conducted, our study is the only one with a late phase (after an average of 15 minutes) to analyze the retention of the contrast in the tubes and its subsequent dispersion in the peritoneal cavity.

Until now, only Volondat *et al*. (2019) and Cipolla *et al*. (2016) undertook a systematic evaluation of other tubal alterations in addition to obstructions. Volondat *et al*. (2019) reported tubal diameter and contrast dispersion in the cavity, while Cipolla *et al*., 2016 evaluated the symmetry of tubal extravasation and the relationship between the position of the tubes and ovaries.

As in previously published studies (Volondat *et al*., 2019; Unterweger *et al*., 2002; Cipolla *et al*. 2016; Sadowski *et al*., 2008), our study demonstrated that the MRI also showed extra-tubal pelvic alterations that were not seen in the HSG-XR (in 40% of our cases), and which were relevant for those patients with infertility: submucous myoma, adenomyosis, endometriosis, micro-polycystic ovaries, and ovaries with signs of low follicle reserve, as well as showing greater detail concerning the adherence process.

When we analyzed only tubes and liquid dispersion in the peritoneal cavity during the HSG-MRI phases and compared them with the HSG-XR, the image aspects were similar in both methods. In the cases in which the HSG-XR was normal, the HSG-MRI also did not identify any alterations. With respect to the other patients (80%) that showed some alteration identified in this phase, we found that:

1-) always, whenever the HSG-XR showed tubal dilation (30%), this finding was also reproduced in the HSG-MRI, being bilateral in all cases; 2-) the findings related to tubal perviousness were identical in both methods, with two patients (20%) having two tubes completely occluded while the other eight (80%) showed pervious tubes; 3-) both methods identified signs of adherence in four patients (40%), without divergences. It is worth noting that the pelvic adherence process was better characterized when we analyzed all of the MRI sequences, enabling a more precise identification of blockage in the vaginal sac, and which structures were adhered, principally in those patients with endometriosis.

With respect to the evaluation of tubal liquid retention in the late phase, in two patients, both methods identified the same finding, including when laterality was considered (in one patient, retention was bilateral, while in the other it was only on the left side). In our first patient in the project, retention was identified only by the HSG-XR and not by the HSG-MRI. This may be explained by the fact that our protocol has yet to be perfected, and with adjustments made over the course of the study, there was a significant improvement in image quality.

In our final patient (tenth) in the study, only the HSG-MRI identified tubal retention. In our opinion, this does not indicate an error in the HSG-MRI, but rather that we achieved an image quality that was so good that the alteration was real and could not be identified by HSG-XR, which is currently considered the gold standard method.

We did not use measures to increase the contrast viscosity, with the aim of facilitating tube visualization, such as combinations with povidone or other contrast mediums with an iodine base; examples of which have previously been proposed in the literature (Unterweger *et al*., 2002; Carrascosa *et al*. 2016; Winter *et al*., 2010). Nevertheless, we were able to visualize the tubes in eight of the nine patients (89%).

The positioning of the cervical catheter in our study was made on the exam table, which probably contributed to a greater success rate compared to that of Unterweger *et al*., (2002), who reported three episodes of dislocation during transport from the preparation room to the exam table, and Volondat *et al*. 2019, with nine dislocations among 40 cases.

Despite the fact that we did not conduct a test to compare pain experienced by the patient during the HSG-XR and the HSG-MRI, the majority of patients reported less intense pain during the HSG-MRI.

Our study had some limitations. Following the initial exam, we perceived the need to implement changes in the protocol as the exams were being conducted, which meant that the first exams followed a protocol that was slightly different in comparison to the last. On the other hand, after making technical adjustments, this process allowed us to obtain high-resolution spatial and temporal images, which enabled better tubal characterization.

We would also highlight that, while spatial and temporal resolution were suboptimal, the first exams still allowed us to analyze tubal patency, contrast dispersion in the peritoneal cavity, and in some cases, even the direct visualization of the tubes, although with less anatomic detail. Another negative aspect was that the diagnostic evaluations of the HSG-XR and HSG-MRI exams, conducted by a single radiologist, were not blind, which could result in a bias in the comparative analysis with other methods. However, as our objective was not to conduct a statistical analysis comparing the two methods, but rather to correlate the findings of HSG-XR with those of HSG-MRI, we believe that this did not have a significant impact on our correlation.

In evaluating the tabulated parameters regarding the tubal characteristics comparing the two methods, we note that the information is similar. Given this, in undergoing a hysterosalpingography exam with magnetic resonance imaging, the patient undergoes one exam at one time with a single preparation, which, in addition to providing all of the usual information of a pelvic MRI, also evaluates the tubes in a detailed and systematic manner without ionized radiation and without iodine contrast.

## CONCLUSION

According to the results of the literature review and our own initial experience, HSG-MRI is promising. We demonstrated an optimized protocol for conducting an HSG-MRI (with excellent image quality), and correlated these with HSG-XR, showing the overlap between the methods. In addition to not being subjected to ionized radiation, patients who have been recommended to have a pelvic MRI as part of a more detailed investigation into infertility can also benefit from undergoing a simultaneous HSG using MRI.

In this way, a simultaneous analysis of the tubes and of pelvic diseases related to infertility can be conducted on the same day and through a single exam, with greater comfort for the patient.
